# Understanding Dalit equity: a critical analysis of primary health care policy discourse of Kerala in the context of ‘Aardram’ mission

**DOI:** 10.1186/s12939-023-01978-4

**Published:** 2023-08-26

**Authors:** Sreenidhi Sreekumar

**Affiliations:** 1 Achutha Menon Centre for Health Science Studies, Sree Chitra Tirunal Institute for Medical Sciences and Technology, Thiruvananthapuram, Kerala India; 2 Sambodhi Research and Communications, Noida, India

**Keywords:** Health equity, Caste, Dalits, Family health centre, Critical discourse analysis

## Abstract

**Background:**

The Government of Kerala in 2017 launched the Aardram Mission with the aim to revamp public health delivery in the State. A key strategy under the mission was its focus on comprehensive primary health care to achieve equitable health care delivery through the Family Health Centre (FHC) initiative. Given this, the current study aims to examine the primary health care policy discourse for their perspectives on caste-driven inequities.

**Methods:**

The study undertook a Critical Discourse Analysis (CDA) of the primary health care policy discourse in Kerala. This included CDA of spoken words by senior health policy actors and policy texts on Aardram Mission and FHC.

**Results:**

Though equity was a major aspirational goal of the Mission, related policy discourse around equity failed to acknowledge caste as a potential axis of health marginalisation in the State. The dismissal of caste manifested in three major ways within the policy discourse. One, the ‘invisibilisation’ of caste-driven inequities through strategies of (un)conscious exclusion of Dalit issues and ‘obliteration’ of caste differences through the construction of abstract and homogenous groups that invisibilise Dalits. Secondly, locating caste as a barrier to primary health care initiatives and health equity in the state, and finally through the maintenance of an ‘apoliticised’ social determinants discourse that fails to recognize the role of caste in shaping health disparities, specifically among Dalits in Kerala.

**Conclusion:**

Given Kerala’s renewed commitment to strengthening its public health provisioning, the acknowledgment of caste-driven inequities is invariable in its path toward health equity and social justice.

## Background

That Dalits (Scheduled Castes or SC in administrative parlance) are one of the most marginalised social groups in contemporary India is not something new to Indian academia and policymakers. While many countries have had similar hierarchisation of societies, none was as complex, pervasive, and long-lasting as the caste system that exists in India. The resilience of caste in the face of myriad historical changes including feudalism, colonial rule, and modern-day capitalism is proof enough of its adaptability and the complex grip it wields over Indian society [1, 2]. This is of crucial relevance in the current study as is the case in any contemporary critique of public policies in India, as caste-based norms remain the dominant structure dictating Indian social organisation through multiple ways [3, 4]. For, caste has had a significant footprint in the overall status of social development in contemporary India and studies on caste-based inequalities suggest that Dalits constitute the most significant proportion of the deprived sections in India [5]. As per the 2011 census, Dalits constitute around 16.6% of the Indian population, a major proportion of which are engaged in the agriculture sector or other low-income jobs and is a social group that has very minimal asset ownership compared to others in India. Dalits also constitute the major section of bonded labourers in India and their literacy status remains 66%, lower than the national average of 73% [6, 7].

### Caste question within Kerala’s developmental paradigm

The Indian state of Kerala despite its low economic performance is often hailed for its achievements within human development indices. The State is often considered a model, even for the third-world regions burdened with the history of colonialist exploitation and chronic developmental inequality. The developmental landscape of Kerala is marked by its impeccable achievements in the social sector reflected as high levels of life expectancy, literacy, and low levels of maternal and infant mortality [8]. An achievement viewed as the product of the state’s long history of social reform movements, agrarian reforms, and land redistribution. While Kerala also boasts of a long history of anti-caste movements and relatively low levels of discriminatory practices based on caste, its development paradigm continues to be riddled with its inability to resolve the growing inequities among its Dalit populations. Dalits constitute around 9% of the state’s population and are characterised by their historically poor indicators of health like low life expectancy, high levels of infant mortality and morbidity rates and poor access to good quality health care, and vulnerability borne out of high levels of out-of-pocket medical expenditure among Dalits in Kerala [9–12].

The continuing presence of health inequities along caste lines in the state is often argued as the product of deep-rooted and historically shaped caste norms leading to social exclusion and denial of opportunities of social development for Dalits including education, landownership, and employment amongst others, despite the strident growth in terms of poverty reduction and human development post the 1980’s in Kerala [13]. The experiences of multidimensional poverty and subsequent lack of access to adequate social and cultural capital among Dalits have also further intensified the caste-based inequities specifically in the context of the neoliberal policy shift towards privatisation in the state [14].

### Kerala Aardram mission and Family Health Centre (FHC) initiative

While Kerala achieved significant improvements over the years in human development indicators and in the domain of health, its achievements in health post the economic liberalisation of the ‘90 s have often been argued as unimpressive. Reflected through the reversal of its achievements within indicators like infant mortality rate, and childhood anaemia during the period between 2003 to 2012 [15]. In addition to this, the state also witnessed a significant rise in incidences of chronic diseases and communicable diseases. Increased marketisation of health and the weakening public health sector has contributed to high levels of out-of-pocket expenditure and subsequent inequities within access to good quality health care at low costs for the underprivileged and marginalised social groups, specifically among the Dalits and Adivasis/Scheduled Tribes (STs) [16, 17]. Kerala known for its ‘good health at low cost’ model increasingly came under pressure and soon transitioned into one of the states with the highest out-of-pocket expenditure for health in the country [18, 19].

In full recognition of the widening gaps within the health landscape in terms of reduced public presence and growing distrust in the public health system, the Government of Kerala initiated the Aardram Mission in 2017 [20]. Aardram mission is part of the 2017 ‘Nava Kerala’ (*translated as* ‘*New Kerala’*) Mission conceived by the incumbent government (led by the Communist Party of India (Marxist)), that sought course corrections in terms of the historical exclusion of various social groups within the state’s developmental trajectory. The Nava Kerala mission within its umbrella consisted of four key sub missions 1) The Aardram mission, 2) Life mission (Livelihood, inclusion, and financial empowerment), 3) mission to revamp public education and 4) ‘Haritha Keralam’ mission (to ensure statewide hygiene, waste management, soil conservation and sustainable farming). These missions were part of the Left government’s aspiration to ensure a developmental model that is much more equitable and inclusive of the underprivileged and marginalised sections in the state [21].

The Aardram mission through its approaches, therefore, also aligned itself with the new national policy roadmap set out by the National Health Policy, 2017 that sought to achieve Universal Health Coverage. These included initiatives such as ‘Ayushman Bharat’ that aimed at the transformation of Primary Health Centres and Sub-Centres as Health and Wellness Centres (HWCs) with the specific aim of delivering Comprehensive Primary Health Care that is easily accessible for communities at the grassroots [22, 23]. On a similar note, the Aardram mission also in view of the UN Sustainable Development Goals (SDG) and to effectively lay out the roadmap for its achievement of SDG-3, ‘Good health and Well-being’, aimed at a comprehensive transformation of existing public health services in the state. Among the key objectives of the mission included strengthening the existing primary health care delivery in the state through the Family Health Centre (FHC) initiative. This entailed the conversion of the existing network of primary health centres across the state as upgraded FHCs that have a revised emphasis on comprehensive primary health care including strategies that ensured preventive and promotive care as well as primary health care approaches focussed on social determinants of health. The mission has been envisaged by the State as its vehicle to overcome its existing burden of rising non-communicable and ageing population and thereby achieve its SDG targets and reduce the rising out-of-pocket health expenditure in the State [24].

The policy affirmation on comprehensive primary health care through the Aardram mission along with the other three missions under the ‘Nava Kerala’ Mission, therefore, presents itself as a unique opportunity for the state to take cognizance of unaddressed health inequity gaps that continue to exist in Kerala. The current study, therefore, locates itself within this context of revived commitment on the part of the Government of Kerala on comprehensive primary health care, reflected through the Aardram and FHC initiative and aims to examine how it problematise the situation of Dalits and the ways to overcome their health challenges.

## Methods

Health policy and systems research (HPSR) has over the years gained substantially from its engagement with the construct of power and its analysis, specifically to gather a nuanced understanding of the mechanisms and structures that generate health inequities and disparities. Application of theories on power and deeper empirical examination of its dimensions does hold significance in the attempts to locate health policies as also a product of wider social, political, and historical contexts and to drive potential revisions and improvements to generate equitable health outcomes through them. Power here is defined as the capacity ‘to do something or act in a particular way’ and ‘to direct or influence the behaviour of others or the course of events. Quite crucial, as power relations do hold the ability to shape societies and societal interactions and thereby also in the process shape health and related policies and subsequent health outcomes. The current study specifically draws from the idea of analysing power and how it may flow through societal expressions in the context of health policies and systems. To analyse how power manifests and expresses itself within health policies reflected through their unique positionalities and expressions of social relations and in turn how they may also engender health disparities [25].

### Critical discourse analysis

One of the suggestive methods to undertake the analytical examination of words both written (policies, laws, news articles, academic texts) and oral (interviews, speeches) is using a Discourse Analysis approach. It facilitates a deeper examination of oral and written acts of communication to bring to the fore the subtle yet shared ways of perceiving the world around us and what may be considered normal [26, 27]. Discourse analysis approaches involve examining language and its use to represent what is being spoken or written about, while also extending its inquiry into what is being omitted and/or assumed which becomes crucial within health policies in terms of their ability to reinforce or sustain status quos that may be socially unjust [28, 29]. Critical Discourse Analysis (CDA) too is drawn from the traditions of discourse analysis in its approach to uncover hidden yet implicit assumptions within language, but in so doing it also extends the practice to resist and reject social inequalities [30]. The dimensions of power, therefore, become crucial within CDA approaches given its preoccupation with the ideas of dominance within the use of language. As is argued by Fairclough, within any order of discourse (health systems in this case), often certain ways of meaning-making are accepted over others making them marginalised or silent [31].

**Fig. 1:**
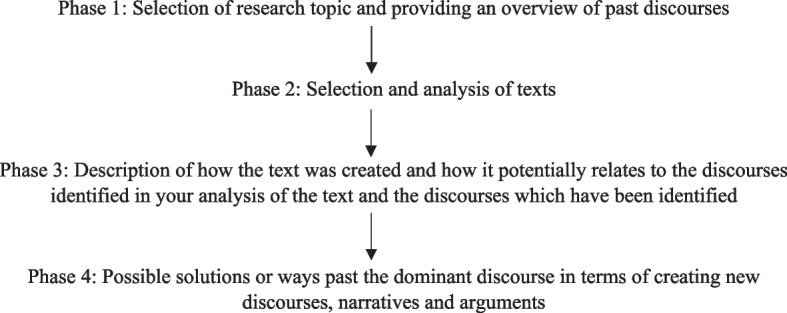
Four phases of CDA

The application of CDA was guided in the current study using the four-phased approach to CDA (Fig. 1) suggested by Cummings et al. [32], beginning with the identification of the question to be productively explored using CDA followed by the selection of texts for analysis, and finally exploring solutions to overcome the dominant discourses (Fig. 1). In light of the 2017 Aardram mission and given its emphasis on addressing the existing health inequalities in the State, the current study aimed specifically the examination of the contemporary policy discourse on primary health care for their inclusion of Dalits as a marginalised social group in Kerala. Given this, the primary material for CDA was prepared from two sources, one the policy documents on Aardram mission as well as that on the FHC initiative by the Department of Health, Government of Kerala. Secondly, through the narratives on primary health care and their ideas related to health marginalisation in Kerala among policy level actors in Kerala. Subsequent to the CDA analysis of the policy discourse on PHC and marginalisation I also discuss the potential ways to overcome the existing dominant discourse that may be exclusive and unfair to Dalit communities in Keralaand influence the creation of fairer alternatives.

The formal policy documents related to Aardram and FHC were sourced from multiple sources. The Aardram document on FHC was collected from the office of the State Health Systems Resource Centre, Department of Health, Government of Kerala in Thiruvananthapuram. In addition, documents on the Aardram mission available from the website of the National Health Mission, Kerala were also sourced. The policy officials’ narratives on primary health care and their ideas on various marginalised communities in Kerala were prepared through in-depth interviews with seniormost policy officials at the Directorate of Health Services (DHS), Department of Health, Government of Kerala (GoK). This involved interviews with officials from the Directorate of Health, District Medical Officers (DMO) as well as District Program Managers (DPM) under the National Health Mission and senior officials of the State Aardram mission (Table 1). The final material for the CDA was prepared by compiling texts from both the formal policy documents on Aardram and FHC as well as by transcribing and translating the interviews with policy officials and uploading them to NVivo™ software for further analysis.

**Table 1:** Details of health policy officials interviewed

Sl.No	Department	Experience in years	Gender	Caste Identity
1	DHS, GoK	31	Female	Non-Dalit
2	DHS, GoK	32	Male	Non-Dalit
3	DHS, GoK	30	Female	Non-Dalit
4	DHS, GoK	29	Male	Non-Dalit
5	DHS, GoK	28	Female	Non-Dalit
6	DHS, GoK	33	Male	Non-Dalit
7	DHS, GoK	26	Female	Non-Dalit
8	DHS, GoK	23	Female	Non-Dalit
9	DHS, GoK	22	Female	Non-Dalit
10	DHS, GoK	28	Male	Non-Dalit
11	State Aardram team	31	Male	Non-Dalit
12	State Aardram team	26	Male	Non-Dalit
13	Ministry of Health, GoK	35	Male	Non-Dalit
14	DHS, GoK	15	Male	Non-Dalit

A central idea within CDA is the fact that social actors from different social locations see and express the world differently and exploring the use of language within discourse could be a way to bring out these differences [33]. This is important as social actions such as discourse hold the power to shape and construct differential and often unjust views of different identities and social processes for its consumers [34]. The current study, therefore, pivots itself on the idea of discursive ‘representation’ of Dalits and caste-driven inequities in Kerala within the Aardram policy and FHC document as well as related narratives of senior health policy officials [32]. To facilitate this the CDA was first guided by a content analysis to identify specific sections of the text that spoke about 1) marginalisation/marginalised communities 2) vulnerability/vulnerable communities 3) community groups requiring priority 3) Scheduled castes/caste 4) Dalits. Undertaking content analysis prior to context analysis is considered to facilitate easier operationalisation of CDA on large bodies of texts and renders the texts conducive for critical interpretation using inductive analysis of selected texts [35]. These were aided through close examination of the specific use of words, discursive strategies adopted, and vocabulary used to represent health-related marginalisation and vulnerability among Dalits or vulnerabilities drawn from caste by policy actors and formal policy texts. In addition, this also involved close examination of the specific contexts in which narratives on caste/Dalits appear as well as the presence or absence of narratives on them. These were expected to glean the different ways in which Dalits are represented within the policy discourse on primary health care as well as the ways in which they may be included or excluded within the discourse on health marginalisation in Kerala.

## Results

The analysis of the policy discourse on primary health care in the current study primarily pivoted itself to the dimension of equity from a caste lens. Specifically, through the examination of how equity and its dimensions are being expressed within the policy discourse on Primary Health Care. This has been undertaken by examining how the policy discourse problematise health vulnerabilities in the state and subsequently examining the nature in which caste is being discussed within the discourse on health inequities in the State. The CDA of the policy discourse in this regard points to three related aspects. Firstly, the discursive strategies adopted by policy actors that aimed at Dalit ‘invisibilisation’ within the policy discourse. These include methods such as the overt exclusion of references to caste as a potential axis of health vulnerability and/or through subtle ways of obliterating caste differences and thereby mobilising health vulnerabilities to inexistent social groupings that further invisibilise Dalits. Secondly to locate caste as a barrier to effective primary health care strategies and thirdly through ‘apoliticised’ discourse on social determinants that is devoid of the consideration to caste-driven health disparities in the State.

### Invisibilisation of Dalits as a marginalised social group

#### ‘Absent presence’ of caste within primary health care policy discourse

A common theme within the discourses on marginalised social groups was the ways in which they carefully avoided references to caste as a probable axis of marginalisation in Kerala, where dimensions of caste remained obvious through their unignorable absence. Discourse that excludes caste-related marginalisation also by extension remain blind to the situation of Dalits in the state and prioritised efforts in the context of primary health care initiatives in the State.

The policy official (Table 2, Quote 1) lists out the various priority groups and presents three categories of health vulnerability in the state, 1) Age-related grouping 2) Disease based grouping (people with chronic diseases and mental health issues) and 3) Health vulnerability based on the social locations of being migrants and tribals. The most dominant theme owing to its frequency of occurrence is the category based on age groups, which is also underscored by reiterating the construct of ‘age groups’ again towards the end of the quote above. A key theme that underlines this discourse is also the ideology that locates health as a raw material for economic productivity. Suggested here through the use of the words like ‘productive life’, ‘able to work’, and ‘reproductive age group’ which seem to shape the policy actors’ ideas on marginalisation. Where people from ‘productive’ age groups are largely considered important and mandate specific priority from a healthcare perspective as they are an important group in ensuring productive communities. Although not concrete, the only occurrence of social identity as a marginalisation category within the above discourse is the references to the tribal populations as well as migrants. However, missing from the narrative were references to caste as well as an acknowledgment of Dalits as a marginalised social group in Kerala. Although the policy narratives allude to its blindness to caste-driven inequities or Dalit inequities by limiting the boundaries of health marginalisation discourse within tribal and coastal communities, there also existed a complacency within the efforts towards addressing health inequities in Kerala (Table 2, Quote 2). Reflected through statements like ‘I don’t think there are groups whose health needs are unaddressed’ while acknowledging tribal and coastal communities as marginalised groups in Kerala. While these narratives on health inequity continues the pattern of caste dismissal among policy actors in the study, the dismissal of caste-driven inequities is also further intensified through a sense of complacency when it comes to having undertaken possible efforts to address health inequities in Kerala.

**Table 2:** Absent presence of Dalits

*Quote 1: “Definitely, there are sections that are vulnerable and need to be prioritised. From the perspective of health, we normally consider ****women within a vulnerable age group who are in their reproductive age groups, children below 5 years****. We also talk about ****old age****, though old age is technically above 60 years, now I feel the minimum age could be increased a little more. Then we can consider ****people with chronic diseases**** who cannot lead a productive life, people with ****mental health issues****, then people who are our guests which are the ****migrants**** and finally ****tribal population****. All these are prioritised by us. That means that all other healthy males and females who are ****able to work**** and within the ****reproductive age group**** are important to us”* (Directorate of Health Service 1)
*Quote 2: “When it comes to marginalised sections in Kerala, there are ****groups like tribal**** where there have been interventions beyond what health department has been doing like social justice department. Then there are also other organisations including NGOs who work amongst tribal communities. If you ask me whether their needs are being addressed, I’d say yes, and they are also the groups whom we generally categorise as marginalised. So, I don’t think there are groups whose health needs are unaddressed in the state or it would be minimal. Because particularly in Kerala where there is very high level of active community involvement there is very little chance of having marginalised groups. Then obviously there is the ****coastal belt**** which is a very long geography for us and where there is a big population too. They too are in a sense a marginalised population”* (Directorate of Health Service 4)

#### Obliteration of caste differences

Dalit exclusion was also found shaped within the policy discourse through their discursive subsumption under abstract and homogenous social categories.

One of the ways in which the policy narratives discursively obliterated caste differences was through the construction of an abstract ‘general community’ and limiting ideas of marginalisation exclusively from the point of geographically and religiously tangible social groups (Table 3, Quote 1). Although the policy narratives repeatedly mentioned tribals as a marginalised group, the reasons for their marginalisation are drawn from the lens of geographical remoteness to health facilities as well as due to tribal communities’ ‘negative’ traits. These included their ‘hard-to-reach’ locations as well as ‘difficult to change’ cultural attributes. The policy narrative also establishes three distinct social groups within the discourse related to marginalisation in the State, through the overt references to tribals, religious groupings and the establishment of a ‘general community’ category. Out of these three, the two marginalised categories are tribals due to their ‘traditionally difficult to change the culture’ and ‘certain religious settlements’. All others are by implication subsumed under the abstract ‘general community’ which also by extension includes various caste groups within the contemporary Kerala diaspora. However, without explicit references to caste as a possible axis of marginalisation the discourse essentially obscures or subsumes the existence of Dalits within this abstract ‘general’ community. The general community is imagined here as a monolithic and homogenous social group that by extension also dismisses all other social class-based disparities including those drawn from caste-hierarchies. This is also reiterated again towards the end of the statement through the categorical assertion being made through the statement ‘other than this we do not have any issue in Kerala’.

**Table 3:** Obliteration of caste differences

*Quote 1: “Marginalised communities, see actually if we think about Kerala, if you ask about such communities, ****definitely tribals****. In tribal areas, they are still remaining as a marginalised section. One of the main reasons for it is our infrastructural changes, one they are living in very remote areas. Then one other reason is that their traditional culture is very difficult to be changed. So, there are definitely these challenges in tribal areas. Otherwise, ****I don’t think there is any marginalised section within the general community****. I haven’t seen from my experience, of course, other than the tribal areas. One more thing is when it comes to immunisation, of course definitely there are ****certain religious settlements****. In such areas, we still have many unimmunised children. In their issue also we haven’t been able to make a change. Other than these, we do not have any issues in Kerala”* (Directorate of Health Service 3)
*Quote 2: “When we speak about marginalised groups, commonly used criteria are geographical or social dimensions, or those who are marginalised within the social pyramid. And above all the ****lower income groups****, or ****tribals in hilly areas****. Tribal groups are marginalised at many levels, be it availability or accessibility of services. Then similarly we have ****urban slums**** or people who live in slum like situations as Kerala does not have any typical slums that one may expect, or ****coastal communities**** also. Then there are groups whom we never used to talk until a few years back, that is ****migrant labourers****, they are also marginalised…. the reasons for urban slums are multiple obviously. One, ****a common reason for the formation of slums are normally economic****, basically unemployed people who come from rural areas or people who come to urban towns as workers of big development activities. Once they complete their work, they tend to remain in urban areas somehow resulting in slums. So, there is a broader socio-political and economic reason behind it and that is a dimension of marginalisation”* (Directorate of Health Service 6)
*Quote 3: “Yes, there are vulnerable sections in our state, where there are gaps in our primary care services. Specifically, in our Adivasi areas or more within ****tribal areas****. Because the primary challenge is the ****hard-to-reach dimension****. By hard-to-reach, I mean areas that are difficult for people to reach physically. All other areas have been encroached upon by people and have been converted as plantation areas and ****Adivasis**** are being pushed to the peripheries. So, the main groups are ****aadivasis, SCST**** (Scheduled Caste Scheduled Tribe) groups are also present in such areas”* (Directorate of Health Service 5)

A second way of discursively obliterating caste differences is through the forceful imposition of logics of economic inequality over caste-driven inequities in Kerala (Table 3, Quote 2). The policy actor within the narrative on social dimensions of health marginalisation talks about four groups in particular, tribal communities, urban slums, coastal communities, and migrant labourers. While the narrative follows the already discussed pattern of failing to recognise caste as a potential axis of health inequity, it also achieves this through its subsumption of Dalit sections through the idea of ‘urban slums’ from a purely economic perspective. While urban slums are seen as a marginalised social group within the narrative, it is also imagined here exclusively from an economic dimension and argued to be a product of rural unemployment leading to urban migration in search of employment and livelihood. This logic, however, runs against the existing evidence that points to the historical links between caste-driven inequities and urban slums, obscuring the current reality that urban slums in Kerala are largely constituted by landless Dalits [36].

Yet another way caste was found subsumed within other categories is through the homogenous category of ‘SC/ST’ (Table 3, Quote 3). The policy narrative in this case mentions the word SC or scheduled caste as located within the larger narrative of scheduled tribes and limits the conversation within the boundaries of tribal communities and related aspects of geographical remoteness and inequities drawn from it. However, by tying together two distinct social groups within a single and homogenous term like SCST, the narrative completely invisibilise the unique challenges faced by Dalit communities and the dimensions of caste-driven social barriers that Dalits face today in the state. Similar pattern of clubbing caste and tribal identities within homogenous categories despite evident differences in terms of their geographical, social, economic, and cultural attributes in Kerala are also noticeable within the FHC document (Table 4).

**Table 4:** Obliteration of caste differences within FHC document

*“The responsibility for arranging the ward level services rest with WHSNC, ASHA, Kudumbasree Health Volunteer, Anganwadi worker, ****SC/ST**** promoter and Arogyasena”* (FHC document, page 10)
*“The role of community health volunteers, ****SC/ST**** promoters, ASHA, Anganwadi worker, Field staff in health services, Staff nurses and Medical Officers in delivering health services should be clearly delineated and the responsibility of each charted out.”* (FHC document, page 62)
*“Colonies, slums, areas inhibited by ****SC/ST****/migrant workers”* (FHC document, page 131)
*“As institutions delivering health care services for the local self- government (LSG) bodies, the FHCs have its tasks cut out. Implementation of the Comprehensive Primary Health Care program of LSGs is one such task, which will see the FHCs working in close coordination with various social sectors including Social Justice, Education, Agriculture, Water supply, ****SC/ST**** development”* (FHC document, page 136)

Within the policy document, the word SC appears 5 times, yet in all these instances it is never used or defined in its own right as a specific social class but always as ‘SC/ST’. Although a possible fallacy in the argument here would be the unique contexts in which these terms may be appearing within the policy document justifying the clubbing of SC and ST. While it may not be completely wrong to argue so, the document however without delineating the unique social realities and health needs of two completely different social groups, such homogenising narratives can only further reinforce the existing discursive patterns of subsuming caste groups under the abstract category of ‘SC/ST’. One that may do justice, neither to Dalits nor to Aadivasis in terms of recognising their differential health needs and thereby pre-empting the possibility of providing equitable primary health care services.

#### Caste as a barrier

To be read along with the patterns of caste invisibilisation achieved through its subsumption within constructed abstract social groups is the perception of caste as a barrier to novel primary healthcare strategies in the State. The discourses on marginalisation largely perceived caste as a fractural construct that can negatively influence the implementation of primary health care strategies, and by implication renders consideration of caste within primary health care modalities as a negative idea.

The narratives related to primary health care innovations often tend to iterate the need to approach the community as a homogenous group (Table 5, Quote 1). However, by forcing a homogeneity lens to communities and its differences, the discourse essentially fails to recognise the relevance of social determinants like caste and even sex. These social groupings are in fact perceived here as negative concepts when undertaking innovations for primary health care. The universally accepted idea of primary health care as a modality that shall be mindful of the unique needs of individuals and social groups to address inequities owing to their social location is lost within this discourse. Specifically, the idea of primary health care vis a vis its objectives of addressing disparities within social groups remains mute by placing caste as a ‘barrier’ to innovate.

**Table 5:** Caste as a barrier

*Quote 1: “Innovations are always new concepts, even if it is an old idea, it will be presented as a fresh concept, that is the key to innovations. So, there is always the barrier of some sections not accepting it, a barrier to getting approval, because always few will not agree. ****A second barrier we can consider is that there are some social determinants****. Suppose when we are developing an innovative program for health, ****we can never consider caste or sex****, or some section of the population. It is always ****difficult to consider only a small section of the community****. So, once we develop and implement an innovation there will always be complaints that some sections did not get representation. So, normally ****new projects on health shall not be constrained by such barriers****”* (Directorate of Health Service 7)
*Quote 2: “When we say about focussing on primary health care, we do not speak about individuals. When we see a well-off family that does not need any support, even if one person from that family becomes incapacitated that whole family will be affected significantly. So, we need to see things from such a perspective. Rather than seeing the individual we should consider the family as a whole……when it comes to healthcare, as we all know it ****does not differentiate between caste or creed****. Health is a concept that is beyond all this and is ****purely a matter of biological concern****. So, if you ask who it affects, it is always an ****individual-level vulnerability****. Even though I am not a person to speak about the relevance of all this, I can only say that ****we should move beyond all this and support all****”* (Directorate of Health Service 2)

The narrative also further considers the exclusion of certain social groups as normalised and a taken-for-granted requirement in the context of primary health care innovations anywhere. This is established through the statement that ‘it is always difficult to consider only a small section’ and ‘there is always the barrier of some sections not accepting innovations.’ This view by extension is also normalising and legitimising exclusions that continue to exist in the case of various marginalised sections of the community including Dalits in Kerala as a given within local primary health care-related projects.

Yet another way caste was excluded and located as a barrier was through the relegation of caste as an irrelevant grouping when it comes to the idea of primary healthcare (Table 5, Quote 2). Erasure of caste and problematising it from a negative vantage point within the policy narratives were also evident when probed for caste as a potential axis of health inequity in Kerala. The narrative examined here starts off with the accepted idea of primary healthcare as not purely individual-centric. However, the policy official’s idea of social groups is restricted here within the narrow definitions of families. The narrative fails to acknowledge and perceive primary health care provisioning on the basis of other social groups like caste or gender amongst others and the differential distribution of health outcomes among them in the state.

However, even this narrow take on social groups evidently turned problematic, upon pointed inquiries of caste as an axis of marginalisation (Table 5, Quote 2). On probing for caste, the policy official goes on to contradict her/his own initial idea of primary healthcare as ‘not individual specific.’ Here disease is being defined purely as a biological phenomenon from a very individualistic perspective. Consideration of caste as a possible axis of inequity is therefore considered irrelevant from such a perspective on health within the narrative. Here too caste is perceived as a negative construct, evident from the value judgment being made in terms of ‘we shall move beyond all this’ difference in matters related to health. The implicit assumption here is that healthcare provisioning shall not be differentiated based on caste identities as diseases after all can affect everyone ‘irrespective of caste or creed’. This is also further reiterated through the emphasis made on the need to provide healthcare equally to all individuals in Kerala. Through the moralistic stance on the need to prioritise equality over equity, the policy official makes a categorical dismissal of caste as a dimension of health inequity and therefore any need to prioritise the differential health needs of Dalits.

#### ‘Apolitical’ discourse on social determinants

The analysis of the State Government’s Aardram Policy document too points to a discourse that is blind to the existing caste-driven disparities in the State. The policy document on Aardram and Family health centre specifically stood out in terms of its unique representation of Dalits and in its complete absence in terms of not recognising caste as an axis of inequity and a social determinant of health marginalisation in the State.

Most striking is the way in which the FHC document locates the word Scheduled caste within its considerations for health care priority (Table 6, Quote 1), specifically in this instance where the policy elaborates the guidelines for implementing comprehensive primary health care. Areas inhabited by scheduled caste are imagined within the policy from the lens of a health hazard that is prone to the emergence of diseases and one that needs to be always under surveillance. Also made evident through its co-location with similarly negative concepts like slums, areas with high mosquito density, sewage treatment plants, and public toilets amongst others. Although the document speaks about social determinants as a major area of impetus the policy never moved beyond such stereotypical representation of Dalits and to locate caste from a social determinants perspective (Table 6, Quote 2). Although the word social determinants appear 32 times within the FHC document, nowhere does it relate the construct of social determinants with disparities among underprivileged social groups (Dalits in this case) and their poor access to health determinants including education, quality housing, sanitation, and livelihood amongst others.

**Table 6:** Apolitical discourse on social determinants of health

*Quote.1: “The field workers should visit the priority areas in each of their day blocks. They should enquire about any emerging public health issues in the locality or institutions. The priority areas are listed below: -*
*1. Areas under the surveillance of WHSNC for control of epidemics*
*2. Areas with high mosquito density*
*3. Colonies, slums, areas inhabited by SC/ST/migrant workers*
*4. BUDS schools/anganwadis/schools*
*5. Correction homes/juvenile homes/children’s homes/orphanages/old age homes*
*6. Areas where public antagonist activities take place*
*7. Sewage treatment plants, water treatment plants, public toilets*
*8. Ward sevakendram if any, present in the area”* (FHC Document, page 131)
*Quote.2:"Transformation in infrastructure, human resources, timings and provision of services along with interventions in social determinants of health would definitely help the state to achieve its sustainable development goals which will ultimately improve the overall health outcome and thus cut down the out-of-pocket expenditure for health”* (FHC document, Health Minister’s message)
*Quote.3:"Different service packages will have to be planned locally for the marginalised and vulnerable population – the differently abled, orphans, the destitute, transgenders, widows, tribal/coastal/urban slum dwellers – in each area” *(FHC document, page 128)

While the policy also suggests the idea of marginalised communities and specific care for them, here too it fails to speak explicitly about social groupings based on caste in its discourse on marginalisation in Kerala (Table 6, Quote 3). The policy uses the word marginalised 6 times, however, nowhere does it mention Dalits or Scheduled Caste as a marginalised group. Through the negation of the idea of caste being a possible societal determinant of health or as an axis of health marginalisation in Kerala, the policy effectively invisibilise and dismiss the issue of Dalits and facilitates the perpetration of the historical marginalisation faced by Dalits in the State.

## Discussion

The Aardram mission and the FHC initiative clearly suggest Kerala Government’s commitment to reviving the primary health care provisioning in the State. This is also reiterated by the fact that it pivots itself on comprehensive primary health care as its approach to achieve the larger goals of equity and social justice for the state. However, the analysis of the policy suggests the persisting gaps in its conceptualisation and articulation of equity and underprivileged specifically from a caste lens. The existing discourse on marginalisation within the primary health care policy in Kerala is characterised by a blindness to caste-driven inequities and the existing social realities of Dalits as a marginalised social group. One that is also curious given its acknowledgment of Adivasis and coastal communities as marginalised, groups which too are historically marginalised in the State [9, 37–39]. The simultaneous presence of Adivasi and coastal communities within the discourse on marginalisation yet unacknowledging the question of caste and Dalit inequities warrants the need to theorise the possible reasons for Dalit invisibilisation. While the discourse on health-related vulnerability and the related dimension of equity examined does suggest the overarching influence of individualistic and person-centric views of health, shaped by a market-driven order of discourse, I believe there is more that is clearly in action when it comes to the discourse on caste and related equity dimensions [40]. Although obvious, the dismissal of caste also, therefore, moves beyond the conventional influence of neoliberal tendencies that are urgent to individualise disease and health outcomes without ever engaging with the larger societal factors determining health disparities across social groups.

A good place to start here would be, therefore, to problematise the nature of representation of caste as a possible axis of marginalisation in Kerala as well as the ways in which the policy engages with Dalits. The primary health care policy discourse examined in the study was evident in its dismissal of the caste question or in recognizing caste as a potential axis of marginalisation. The policy narratives were characteristically evident with an ominous reluctance to mention the word caste or scheduled castes related to discourses on marginalisation and health inequity in the state. Beyond the recognition of Adivasi and coastal communities, the marginalisation narratives in the current study almost exclusively existed along the axes of biomedical markers of age and sex, access to drinking water, and hygiene. The palpable need to dismiss any reference to caste within marginalisation discourses within the current study alludes to a conscious or unconscious yet strong repugnance towards discussions on caste. Quite similar to what sociologist Satish Deshpande argued in his thesis on ‘castelessness’, where he discusses how various historical legal, and policy decisions on the issue of caste and reservation in India have shaped a new ‘common sense’ of caste in India. He argues that the word caste is almost often immediately reinterpreted as ‘lower castes’ within the collective discourses. This also has parallels to similar recontextualizations within other universal categories of gender and race. Gender is almost often construed as women from a conventional patriarchal common sense and race as people of colour from a white supremacist sense [41].

The reductionist logic of caste as ‘lower caste’ has significant relevance in the current study too, since dismissing caste also by extension suggests the exclusion of Dalits themselves as a marginalised social category. Through the avoidance of discussions on caste, there is an uncanny resemblance of the Brahminic logics of purity and impurity being carried over to the realm of social discourses on health and by extension exclusion of Dalits. An evident contempt to even discuss caste among the ruling elites, leading to the existing caste blindness within policies. This is also in line with the ‘Social Nausea’ argument by sociologist Awanish Kumar, where he argues that Indian policy today suffers from the potent combination of social isolation backed by the regressive notion of Brahminical caste and that of social nausea of elite castes towards utterances of caste and Dalits. The result is the inability of public policies on health to effectively address the real issues of the masses in India [42]. The fact that one can discern a similar ‘nausea’ towards utterances of caste within the examined policy discourse is also not helped by the current nature of the health system policy landscape in the state. The realm of senior health policy officials in Kerala is constituted mostly by non-Dalits barring a few Dalit officials as observed during the study. A phenomenon that extends beyond the state boundaries, as the health system and its manpower in India is also similarly characterised by the significant underrepresentation of Dalit sections and overwhelmingly dominated by non-Dalits despite policies of affirmative action. Often also argued to be one of the key reasons for the existence of healthcare policies and provisioning that remains unfair to Dalits in the country [4, 43–45].

The discursive ostracization of Dalits also seems to have been facilitated by the differences in the geographical attributes of Aadivasis/coastal communities and Dalits in Kerala. Historically Aadivasi and fisherfolk communities have remained distinct from their non-Aadivasi/coastal counterparts in Kerala. Specifically, in terms of the almost exclusive geographical presence of Aadivasi communities along the Western Ghats and fisherfolks within the long coastal belts of Kerala with their own distinct socio-cultural identities [46, 47], one that is also repeated frequently within the policy narratives in the study. Dalits, however, don’t really present in the strictest sense a geographical distinction that makes them stand apart from non-Dalits except for the fact that landless Dalits are accommodated in contemporary Kerala in 26,198 ‘colonies’ across the length and breadth of the state, marked by their abject poverty [4]. Chronic health challenges of Aadivasis and coastal communities, therefore, become relatively difficult to silence within policy discourse on health equity in Kerala owing to their discernibly distinct spatio-cultural patterns. On the contrary, Dalits given their lack of any popularly tangible spatio-cultural distinctions are easier to discursively invisibilise using ‘Savarna’ strategies of constructing abstract categories or limiting the issue within economic dimensions that are shorn off the social effects of caste segregation.

What’s also not helping is the adversarial position historically adopted by the political left including Communist parties in India and in Kerala towards caste and caste-identity politics. The urgency in declaring caste-identity politics as an antithetical force to Marxian class politics has often contributed to the political left’s inability to accommodate the struggles of various historically marginalised social groups in India including Dalits [1]. The subsumption of caste from an economic lens manifested here through the discursive practices like considering ‘urban slums’ from an exclusively economic perspective, are characteristic extension of the political position held historically by Communist parties in Kerala. However, this obfuscates the deep-rooted links between caste-driven inequities and subsequent economic impoverishment of Dalits in contemporary Kerala. Based on the 6^th^ report of the Administrative Reforms Commission of the Kerala government, majority of the state’s slum population is constituted by marginalised Dalit sections and is characterised by poor housing status, subpar infrastructural facilities, and sanitation services amongst a host of others [36]. Besides urban slums, a crucial fact in the context of Dalits and related developmental inequity is their formal government-sanctioned ghettoization in Kerala. Exclusively owing to their caste status Dalits in Kerala have over the years forced to live within demarcated land areas, often known as ‘SC colonies’. Out of the total Dalit population of 9.1% in Kerala, 7.9% are still ghettoised within such demarcated colonies by the government [4]. The researcher’s own findings from informal observations of various village panchayats in Kerala and observations of Dalit colonies as well as existing evidence on the status of Dalits in Kerala too suggest this pattern. Dalit neighbourhoods in Kerala are often marked by their limited land availability. More than single Dalit households are located within a small area of under 2–3 cents. The Dalit neighbourhoods often also face the issue of limited availability of running water. Owing to these conditions, safe drinking water and waste disposal remain key areas of concern drawn from their social location of caste in Kerala [44]. However, by failing to acknowledge these pathways of economic impoverishment shaped by caste identities within a health policy like Aardram mission, the primary health care policy the state continues to sustain the tradition of caste exclusive developmental discourse in the state.

An obvious corollary being the stark contradictions within the state’s health policy that is adamantly caste blind despite chronic disparities both in terms of access to basic determinants of health as well as health outcomes among Dalits. A toxic contrast, arguably drawn from the ‘Savarna (upper caste) gaze’ that is relentless in its attempt to normalize Dalit invisibilisation within its discourse on health inequities. Achieved in this case through clandestine ways of shaping discourse on health inequities that is quick to deny the spotlight on caste-inequities either through dismissal or by ‘mobilising health vulnerabilities’ to amorphous social groupings that shall never have a Dalit focus as its priority. A rather lucid demonstration of how caste continuously arranges and rearrange itself to find ways and means to continue its status quo and maintain the social order of caste hierarchies in Indian societies [45]. These patterns allude to a health system discourse in Kerala whose gaze is fundamentally casteist and sustains this caste ‘enculturalisation’ within its policy discourse on health through multiple intersecting factors. Borrowing Althusser, the health policy discourse that is blind to the realities of caste in Kerala signifies a ‘contradiction’ or an unjust and dominant ‘social formation’ brought into existence by an ensemble of ‘uneven developments’ [48]. Uneven developments including but not limited to those ranging from the influence of macro-level neoliberal discourse on healthcare policies to more meso/micro level contextual aspects like power asymmetries between Dalits and non-Dalits in shaping developmental discourse, unfair representation of Dalits within positions of power, dominant political ideologies and unequal growth opportunities available for Dalits in Kerala, all of which has its own historical contexts and distinct ways of emergence in Kerala.

I, therefore, argue that as a first step it is important to deconstruct current policy discourses on development in the state by carefully breaking them down to their individual contradictions that constitute their exclusive positions and subjecting them to deeper understanding and subsequent problematisation [49]. Insights to this could be drawn from the rich traditions of post-colonial critiques that aims to dismantle the infamous ‘white gaze’ within Western developmental discourse in an attempt to similarly call out and problematise the ‘Savarna gaze’ within Indian development discourse [50]. A committed effort to speak into existence the caste contradictions manifest as systematised exclusion of Dalits and caste-driven power asymmetries within knowledge creation spaces in India, an objective implicit also within the current study. Discursive approaches of research reflexive of caste dimensions within policy discourse analysis are therefore a first step in this attempt to shape fair alternatives to dominant discourses that create and recreate unfair social practices and systems.

## Conclusion

While there is a renewed rhetoric against health inequities under the Aardram mission and FHC initiative, the Kerala health system’s policy discourse on primary health care suggests the persisting presence of a ‘Savarna gaze’ that is quick to dismiss the role of caste-related social hierarchies in shaping health disparities among Dalits in the state. Through a discourse that is silent about existing caste-driven health inequities, the renewed policies on comprehensive primary health care under the FHC initiative also stand to engender social practices of primary health care at the grassroots in the state through various Local Self-Government Institutions (LSGIs) that are also similarly blind to caste inequities. This is crucial as a key anchor point for the implementation of the Aardram mission and FHC initiative is the ability of decentralised LSGIs across Kerala to pro-actively undertake and implement the strategies envisioned through the mission at the level of communities at the grassroots [51]. The policy neglect of Dalits is therefore deeply problematic and requires an urgent re-examination and course correction of its views towards health inequities in the state and the related actions to resolve them, through more caste-reflexive approaches. I believe only then can a policy like Aardram can ever achieve its larger goals of social justice and equity for all in Kerala.

## Data Availability

The data that support the findings of this study are available from the corresponding author of the study on reasonable request.

## Abbreviations

SDG: Sustainable Development Goals
SC: Scheduled Caste
ST: Scheduled Tribe
UN: United Nations
FHC: Family Health Centre
HPSR: Health Policy and Systems Research
CDA: Critical Discourse Analysis
LSGI: Local Self-Government Institutions

## References

[1] Teltumbde A (2016). Dichotomisation of caste and class. Econ Pol Wkly.

[2] Bhardwaj P, Sharma V (2020). Untouchability-the Plight of Dalits: in the Works of Dalit Writers, Dr. B.R. Ambedkar and Om Prakash Valmiki. Int J Res -GRANTHAALAYAH.

[3] What is India’s caste system? - BBC News. 2019. Available from: https://www.bbc.com/news/world-asia-india-35650616. Cited 2022 Jan 12.

[4] Pramod M (2020). As a Dalit Women. CASTE / A Global J Soc Exclusion.

[5] Sundaram K, Tendulkar SD (2003). Poverty among Social and Economic Groups in India in 1990s. Econ Polit Wkly.

[6] Census of India: Primary Census Abstract. 2011. Available from: https://censusindia.gov.in/pca/default.aspx. Cited 2022 Jan 12.

[7] Pankaj AK (2019). Caste and discrimination in welfare: social exclusion of Dalits in Uttar Pradesh. Contemp Voice Dalit.

[8] Franke RW, Chasin BH (1992). Kerala State, India: radical reform as development. Int J Health Serv.

[9] Navaneetham K, Kabir M, Krishnakumar CS (2009). Morbidity patterns in Kerala: levels and determinants.

[10] Mukherjee S, Haddad S, Narayana D. Social class related inequalities in household health expenditure and economic burden: evidence from Kerala, south India. Int J Equity Health. 2011;10:1-13. 10.1186/1475-9276-10-1. 10.1186/1475-9276-10-1 PMC3024220 21214941

[11] Kannan KP (1991). Health and development in rural Kerala: a study of the linkages between socioeconomic status and health status.

[12] Census India (2011). Kerala Population-Census India 2011.

[13] Thresia CU (2018). Health inequalities in South Asia at the launch of sustainable development goals: Exclusions in Health in Kerala, India need political interventions. Int J Health Serv.

[14] Synergy: The Journal of Contemporary Asian Studies. Navigating Caste Inequality in Kerala: Caste as Present, Hidden, and Denied. 2018. Available from: https://utsynergyjournal.org/2018/04/20/navigating-caste-inequality-in-kerala-caste-as-present-hidden-and-denied/. Cited 2023 May 24.

[15] National Family Health Survey, India (NFHS), second (1998–1999), and third (2005–2006) rounds. Available from: http://rchiips.org/nfhs/nfhs3.shtml. Cited 2023 Apr 14.

[16] Thresia CU (2014). Social Inequities and Exclusions in Kerala’s ‘Egalitarian’ Development. Monthly Review.

[17] Thresia CU (2013). Rising private sector and falling “good health at low cost”: health challenges in China, Sri Lanka, and Indian state of Kerala. Int J Health Serv.

[18] PHCPI (2015). Kerala, India: Governance.

[19] Bhatia P (2015). Kerala has the highest out-of-pocket expenditure on healthcare. Medical Dialogues.

[20] Government of Kerala. NavaKeralam Karmapadhai | Local Self Government Department. 2017. Available from: http://lsgkerala.gov.in/en/resources/Nava-Keralam. Cited 2022 Nov 14.

[21] Local Self Government Department G of K (2017). NavaKeralam Karmapadhathi.

[22] Lahariya C. “Ayushman Bharat” Program and Universal Health Coverage in India - PubMed. Indian Pediatr. 2018. Available from: https://pubmed.ncbi.nlm.nih.gov/29978817/#full-view-affiliation-1. Cited 2023 Jun 1. 29978817

[23] Lahariya C (2020). Health & wellness centers to strengthen primary health care in India: Concept, progress and ways forward. Indian J Pediatr.

[24] State Health Systems Resource Centre Kerala (2019). Comprehensive primary health care through family health centres.

[25] Topp SM, Schaaf M, Sriram V, Scott K, Dalglish SL, Nelson EM (2021). Power analysis in health policy and systems research: A guide to research conceptualisation. BMJ Glob Health.

[26] Steel EJ (2019). The duplicity of choice and empowerment: Disability rights diluted in Australia’s policies on assistive technology. Societies.

[27] Sacks H. Lectures on conversation: Volume I. Malden: Blackwell; 1992.

[28] Sieleunou I, Turcotte-Tremblay AM, Fotso JCT, Tamga DM, Yumo HA, Kouokam E (2017). Setting performance-based financing in the health sector agenda: A case study in Cameroon. Global Health.

[29] Narayanan SP, Rath H, Mahapatra S, Mahakur M. Preparedness toward participation in disaster management: An online survey among dental practitioners in a disaster‑prone region of Eastern India. J Educ Health Promot. 2023;13(2):1–11. 10.4103/jehp.jehp_914_22 PMC10127487 37113417

[30] Schiffrin D, Tannen D, Hamilton HE (2007). The handbook of discourse analysis.

[31] Handford M, Gee JP, editors. The Routledge handbook of discourse analysis. routledge; 2013 Jun 17.

[32] Cummings S, De Haan L, Seferiadis AA. How to use critical discourse analysis for policy analysis: a guideline for policymakers and other professionals. Knowledge Manage Dev J. 2020;15(1):99–108

[33] Fairclough N, Wodak R, Meyer M (2001). Critical discourse analysis as a method in social scientific research. Methods of critical discourse analysis.

[34] Chilton P. Missing links in mainstream CDA: Modules, blends and the critical instinct. In R. Wodak, P. Chilcote (Eds.). In: A new agenda in (critical) discourse analysis. John Benjamins Publishing Company; 2005. p. 19–51. Available from: https://benjamins.com/catalog/dapsac.13.05chi. Cited 2023 Jun 1.

[35] Mengibar AC (2015). Critical discourse analysis in the study of representation, identity politics and power relations: A multi-method approach. Commun Soc.

[36] Madhyamam. Slum and colony dwellers in Kerala faced with bad living conditions,says Administrative Reforms Commission report. 2020. Available from: https://english.madhyamam.com/kerala/slum-and-colony-dwellers-in-kerala-faced-with-bad-living-conditionssays-administrative-reforms-commission-report-600847. Cited 2023 May 25.

[37] Haddad S, Mohindra KS, Siekmans K, Mk G, Narayana D (2012). “health divide” between indigenous and non-indigenous populations in Kerala, India: Population based study. BMC Public Health.

[38] Narain JP (2019). Health of tribal populations in India: How long can we afford to neglect?. Indian J Med Res.

[39] Ulahannan SK, Srinivas PN, Sreekumar S, Jament J, Mohan M (2022). COVID-19 and Multiple Inequalities The Case of a Coastal Community in Kerala. Econ Polit Wkly.

[40] Hänninen S, Lehtelä KM, Saikkonen P (2019). The Relational Nordic Welfare State: Between Utopia and Ideology.

[41] Deshpande S (2013). Caste and Castelessness in the Indian Republic: Towards a Biography of the ‘General Category’. Rev Dev Change.

[42] Kumar A (2020). Reading ambedkar in the time of covid-19. Econ Polit Wkly.

[43] George S. Caste and care: is Indian healthcare delivery system favourable for Dalits? Institute for Social and Economic Change; 2015.

[44] Maktoob. Chalo Thiruvananthapuram ; Wipe Out Caste Colonies. Reconstruct the Kerala Model. 2023. Available from: https://maktoobmedia.com/india/319-2/. Cited 2023 May 25.

[45] Sheth DL. School of Advanced Study, University of London. 2011 [cited 2023 Aug 25]. Caste and Exclusion: Issues of Theory and Policy. Available from: https://sas-space.sas.ac.uk/5663/1/AHRC_15%2C_DL_Sheth%2CCaste_an_Exclusion_of_Theory_and_Policy_dated_11_may_201_new%5B1%5D.pdf.

[46] Government of Kerala. KIRTADS. Tribals in Kerala. 2016. Available from: https://kirtads.kerala.gov.in/tribals-in-kerala/. Cited 2023 May 29.

[47] Lisba Yesudas, Johnson Jament. Terralingua. Marine Biodiversity & Cultural Diversity: The Coastal Communities of Trivandrum, Kerala, India. 2019. Available from: https://terralingua.org/langscape_articles/marine-biodiversity-cultural-diversity-in-the-coastal-communities-of-trivandrum-kerala-india-iysc/. Cited 2023 May 29.

[48] Althusser L (1969). For Marx.

[49] Resch RP (1992). Althusser and the Renewal of Marxist Social Theory.

[50] Bilgen A, Nasir A, Schöneberg J (2021). Why positionalities matter: reflections on power, hierarchy, and knowledges in “development” research. Can J Dev Stud.

[51] Krishnan A, Varma RP, Kamala R, Anju R, Vijayakumar K, Sadanandan R (2023). Re-engineering primary healthcare in Kerala. Public Health Action.

